# Meningococcal Pneumonia in a Patient With Pulmonary Amyloidosis and a Recent COVID-19 Infection

**DOI:** 10.7759/cureus.68840

**Published:** 2024-09-06

**Authors:** Kazuhisa Konishi, Toshiyuki Tanaka, Kohei Yamamoto, Taisuke Tsuji, Chieko Takumi

**Affiliations:** 1 Respiratory Medicine, Japanese Red Cross Society Kyoto Daiichi Hospital, Kyoto, JPN

**Keywords:** covid-19, covid associated pneumonia, neisseria meningitidis, pulmonary amyloidosis, respiratory disease

## Abstract

A 64-year-old man visited our outpatient clinic with chief complaints of high fever and throat pain. His medical history was significant for pulmonary amyloidosis that was observed at our outpatient clinic, and his recent recovery from COVID-19. Findings from imaging studies included thickening of the bronchial walls, infiltrates of the left upper lobe, and pre-existing pulmonary nodules from amyloidosis. A peripheral blood examination revealed leukocytosis and elevated C-reactive protein levels. His signs and symptoms suggested bronchopneumonia and antimicrobial treatment was initiated. Sputum microscopic examination revealed gram-negative cocci and culture growth revealed to be *Neisseria meningitidis*, with follow-up bacterial specimens after treatment demonstrating diminished microbes. Despite a medical history of amyloidosis and COVID-19, the patient’s clinical course resulted in favorable outcomes. The *N. meningitidis* infection is a rare condition in generally healthy individuals, and certain conditions may be related to the contraction of the pathogen, otherwise seen primarily in immunocompromised hosts. In our case, the medical history of amyloidosis and recent COVID-19 infection may have contributed to the development of meningococcal bronchopneumonia.

## Introduction

*Neisseria meningitidis* is a contagious pathogen that spreads via respiratory droplets through patient contact. It can potentially cause medical emergencies, and prompt diagnosis and treatment with antibiotics are essential to prevent serious complications [1]. Most meningococcal infections occur in special conditions, such as within incarcerated conditions or among immunocompromised hosts [2,3]. However, other conditions that increase susceptibility to meningococcal infections may be overlooked. Here, we report a case of meningococcal bronchopneumonia in a patient with a history of pulmonary amyloidosis and recent recovery from COVID-19.

## Case presentation

**Figure 1:**
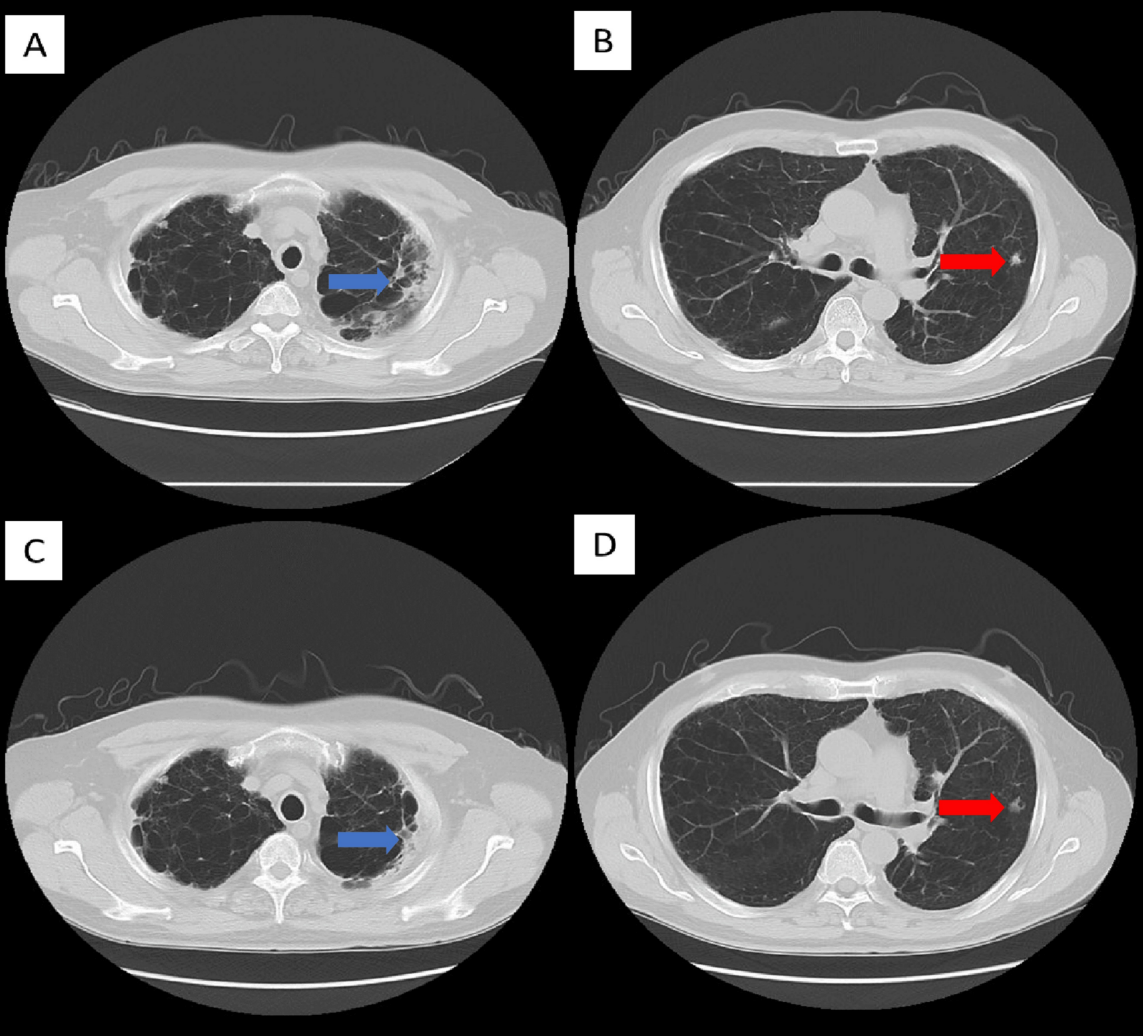
The CT scans of the patient at the time of admission (A, B) and after recovery (C, D) The images demonstrate that infiltrates from meningococcal pneumonia improved with treatment (blue arrows) and there were persistent findings of amyloid nodules (red arrows). Background emphysematous changes were also evident.

The patient was a 64-year-old man with a 30-pack-year smoking history. He was referred from a primary care clinic with symptoms of a high fever of over 39 °C and decreased oxygen saturation of 89% at room air. His medical history was significant for pulmonary amyloidosis with light chain (AL) amyloid deposits confirmed by surgical biopsy and hepatitis C treated with pegylated interferon and ribavirin, resulting in a sustained virologic response. He has received follow-up observations for lung lesions every six months at our outpatient clinic, with stable general and radiological findings. He had also contracted COVID-19 four months prior to his referral. He was treated for COVID-19 as an outpatient without administration of systemic steroids, resulting in a good recovery. His chief complaints upon referral from a primary care physician included high fever, throat pain, and rust-colored sputum. A CT scan revealed edematous changes in the tracheal walls and consolidations in the left upper lobe, as well as smooth-edged nodules in multiple lobes of the lung from his pre-existing condition of pulmonary amyloidosis (Figure 1 A and B).

**Figure 2:**
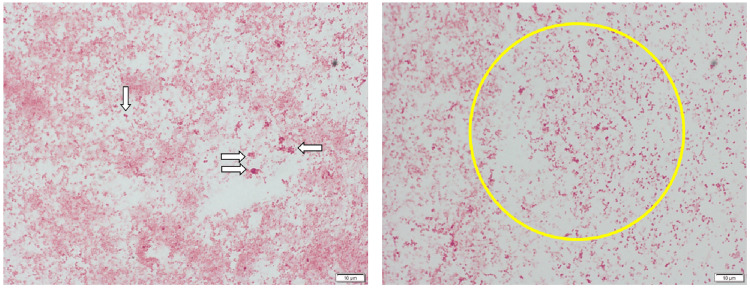
Sputum Gram stains of the patient at admission Sputum Gram stain smears demonstrate gram-negative cocci in clusters (arrows, left image; yellow circle, right image). Cultures were revealed to be positive for *N. meningitidis*.

Peripheral blood revealed leukocytosis with increased neutrophils and elevated C-reactive protein levels (Table 1). The patient was diagnosed with bronchopneumonia and admitted to our hospital. Antimicrobial treatment with sulbactam/ampicillin (3 g every eight hours) and intravenous fluid replenishment was initiated. The patient's clinical signs and symptoms improved promptly after the treatment. On admission, his sputum Gram stain was positive for gram-positive cocci, and cultures representing the lower respiratory tract revealed *N. meningitidis*. In contrast, nasal swab cultures were negative for microbial findings, establishing a clinical diagnosis of meningococcal bronchopneumonia (Figure 2).

**Table 1:** Peripheral blood findings at the time of admission

Test items	Values	Normal range
White blood cells	13260 /uL	4000 - 8000
Red blood cells	4.9×10^6^ /uL	4.27 - 5.70
Hemoglobin	14.9 g/dL	13.5 - 17.6
Hematocrit	45.80%	39.8 - 51.8
Platelet	7.4×10^4^ /uL	15×10^4^ - 35×10^4^
Mean corpuscular volume	93.5 fl	82.1 - 101.6
Mean corpuscular hemoglobin	30.4 pg	28.0 - 34.6
Mean corpuscular hemoglobin concentration	32.50%	31.6 - 36.6
Basophils	0.20%	0.0 - 2.0
Eosinophils	0%	0.0 - 8.0
Neutrophils	73.90%	40 - 75
Lymphocytes	17.70%	15 - 45
Monocytes	8.20%	2 - 10
Total bilirubin	2.1 mg/dL	0.3 - 1.2
Aspartate aminotransferase	23 U/L	13 - 33
Alanine transaminase	16 U/L	8 - 42
Lactate dehydrogenase	200 U/L	124 - 222
Glucose	189 mg/dL	70 - 140
Blood urea nitrogen	15 mg/dL	8 - 22
Creatinine	1.00 mg/dL	0.65 - 1.07
Sodium	137 mEq/L	138 - 146
Pottasium	3.9 mEq/L	3.6 - 4.9
Chloride	101 mEq/L	99 - 109
C-reactive protein	13.87 mg/dL	0 - 0.30

The patient was not systemically immunocompromised and denied being incarcerated or in contact with another individual with an established diagnosis of meningococcal infection. His clinical course was generally favorable, with his body temperature becoming afebrile on the day following admission, and his follow-up sputum culture results were negative for pathogens. Blood cultures obtained at the time of referral and four days after admission were also negative for microbes. The patient was discharged 7 days after emergency admission. Follow-up CT demonstrated improved findings for bronchial edematous changes, while nodules of AL amyloidosis persisted (Figure 1 C and D). Currently, the patient denies the development of any neurological complications and continues to attend follow-up visits at our outpatient clinic.

## Discussion

Here, we report a case of meningococcal pneumonia with favorable outcomes. Prompt treatment with antibiotics results in a limited disease without aggressiveness. Follow-up outpatient visits confirmed that the infection was localized to the respiratory system and was otherwise noninvasive, demonstrating a stationary overall condition. The patient continues to receive outpatient observations for a pre-existing condition of pulmonary amyloidosis.

A few features are worth noting in the development of meningococcal pneumonia. The patient’s history of pulmonary amyloidosis is a potentially pertinent attribute of *N. meningitidis* contraction. In amyloidosis, amyloid protein deposits are derived from immunoglobulin light chains, which are components of antibodies. These abnormal proteins are mostly derived from plasma cells [4]. The accumulation of amyloid deposits can interfere with the normal function of the affected organs, leading to possible complications with infections, including pneumonia, possibly occurring in patients with amyloidosis. The proposed cellular mechanism for susceptibility to infection in patients with amyloidosis includes serum amyloid components binding to the meningococcal pathogen's cell membranes, demonstrating a powerful antiopsonic effect both in vitro and in vivo. These results suggest that the accumulation of amyloids results in reduced phagocytosis and killing of the bacteria [5]. Therefore, patients with a pre-existing diagnosis of pulmonary amyloidosis may be predisposed to meningeal pathogens, which may partly explain the infection with Neisseria species in this particular case.

The patient's recent history of COVID-19 is another important factor to consider when discussing the relevance of meningococcal pneumonia. Numerous reports have suggested that the preceding COVID-19 respiratory system infection is associated with bacterial co-infection [6,7]. In general, viral damage disrupts the barrier function of epithelial cells, and the subsequent entry of bacteria into the respiratory tract may further cause damage by inhibiting the repair and regeneration of the epithelial cell layer [8]. Although whether this mechanism is specifically applicable to SARS-CoV-2 and *Neisseria spp.* remains to be determined, patients with COVID-19 can develop bacterial co-infections from a broad range of organisms [9].

Risk factors for an increase in the pathogenesis of invasive *N. meningitidis* infections include immunodeficiency or special conditions such as incarceration. In our case, the patient was generally healthy in terms of activities of daily living but had a complicated medical history of amyloidosis. His recent COVID-19 contraction is another possible immune-modulating event. These conditions may be predisposing factors for meningeal infections.

One major limitation of our report is that the methods used to assess changes in the immune status prior to and after COVID-19 infection can be challenging, and there may have been other factors that may have contributed to the *N. meningitidis* infection that developed in this patient. While clinical trials to assess susceptibility to *N. meningitidis* in patients with AL amyloidosis can provide definitive answers, the scarcity of these conditions combined can make it challenging to conduct a study. Careful microbial evaluation in acute settings is essential to collect clinical cases and evaluate possible interactions between *Neisseria spp.* and accumulated amyloid in lung tissues [10].

## Conclusions

We encountered a case of meningococcal infection of the respiratory tract in a patient with a history of AL amyloidosis and a recent COVID-19 infection. The meningococcal infection was limited to the respiratory tract and was not considered invasive. The patient's clinical status improved with the prompt administration of antibiotics, and he was cured without any complications. Although he was considered a generally healthy individual with AL amyloidosis, his recent COVID-19 infection may have been a predisposing factor for contracting *N. meningitidis*.
